# Supplementary material for: Laser scanning reflection-matrix microscopy for aberration-free imaging through intact mouse skull
Source: Nat Commun. 2020 Nov 12;11:5721. doi: 10.1038/s41467-020-19550-x (PMC7665219; doi:10.1038/s41467-020-19550-x)
Supplement: Supplementary file 1 — Supplementary Information [file 41467_2020_19550_MOESM1_ESM.docx]

**Supplementary Information**

**Laser scanning reflection-matrix microscopy for aberration-free imaging through intact mouse skull**

Seokchan Yoon^1,2,+^, Hojun Lee^1,2,+^, Jin Hee Hong^1,2^, Yong-Sik Lim^3^ and Wonshik Choi^1,2,*^

^1^*Center for Molecular Spectroscopy and Dynamics, Institute for Basic Science, Seoul 02841, Korea*

^2^*Department of Physics, Korea University, Seoul 02855, Korea*

^3^*Department of Nano Science and Mechanical Engineering and Nanotechnology Research Center, Konkuk University, Chungbuk, Korea.*

*^+^These authors contributed equally to this work.*

[^*^*wonshik@korea.ac.kr*](mailto:*wonshik@korea.ac.kr)

# Supplementary Note 1: Detailed experimental setup


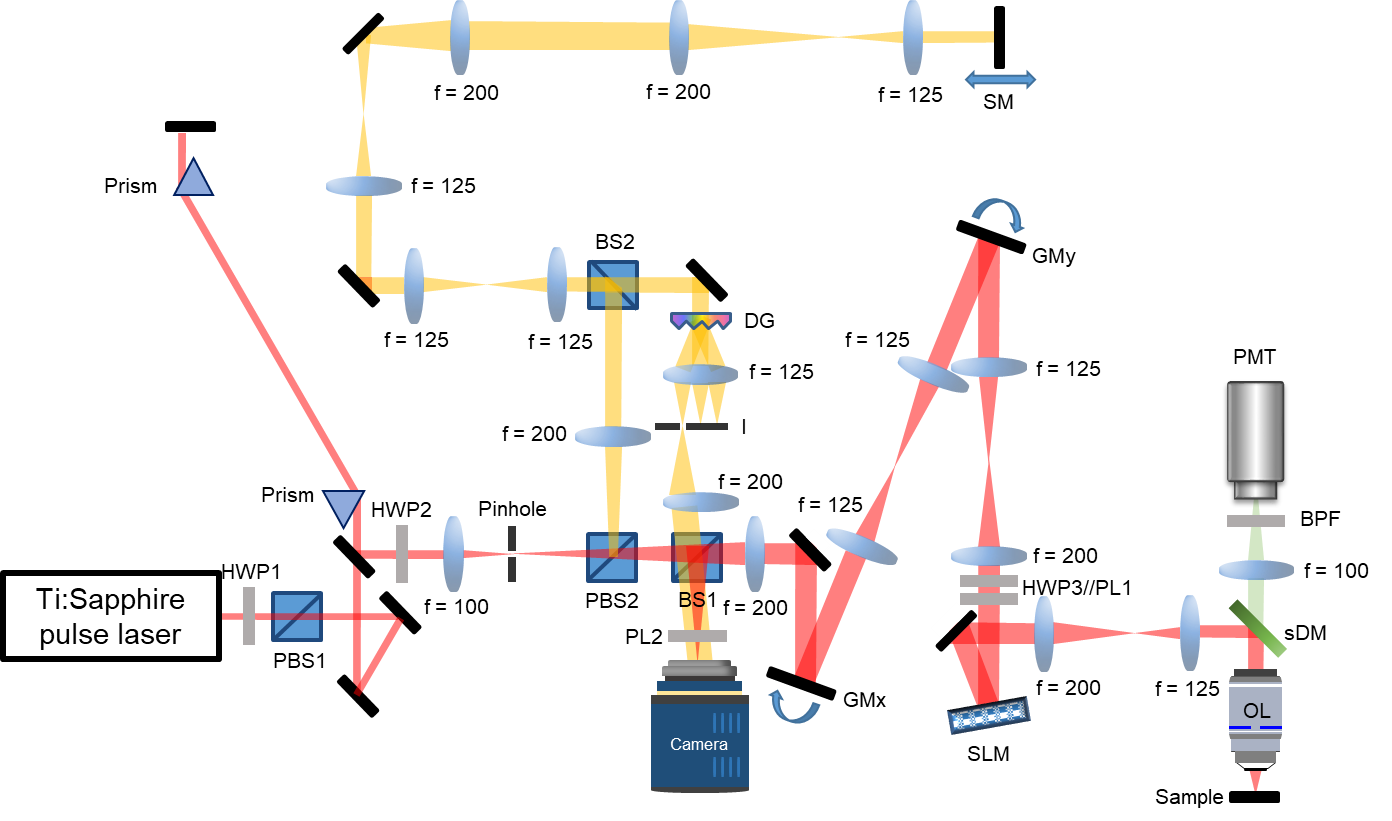


**Supplementary Figure 1. Details of experimental setup.** HWP: half wave plate, PBS: polarizing beam splitter, BS: beam splitter, GMx and GMy: galvometer scanning mirrors, PL: polarizer, SLM: spatial light modulator, sDM: short-pass dichroic mirror, OL: objective lens, BPF: band pass filter, PMT: photomultiplier tube, SM: scanning mirror, DG: diffraction grating, f: focal length of lens in mm.

The detailed schematic of the LS-RMM setup is shown in Supplementary Figure 1. A Ti:Sapphire pulsed laser (center wavelength: 900 nm, bandwidth: 25 nm) was used as a low-coherence light source. HWP1 and PBS1 were used to adjust the intensity of the beam sent to the sample. The prism pair was used for compensating dispersion induced by the beam delivery optics and the objective lens in the sample beam. HWP2 was inserted to adjust the power ratio between the sample (red) and reference (orange) beams split by PBS2. In the sample path, two galvo mirrors (GMx and GMy) were installed to raster scan the focal spot of the beam at the sample surface. In addition, the SLM was inserted in order to physically correct aberrations. The SLM was used as a flat mirror in the case of CLASS imaging. The sample beam was then focused on the sample by the objective lens (Nikon, x60, NA 1.0). Backscattered waves from the sample were collected by the objective lens and reflected by BS1 to be recorded at the CMOS camera (Pco Edge 4.2) positioned on the conjugate image plane. More often than not, there are stray reflections from the optics in the sample arm. Most of the noise can be removed by properly adjusting the axes of HWP3, PL1 and PL2. The reflected beam by PBS2 was used as a reference planar wave to measure the complex electric-field map of the sample beam arriving at the camera. To ensure that the interference occurs between two beams over the entire camera plane, the first-order diffraction by the diffraction grating (DG: Edmund, 110 lp/mm) was used as a reference wave. By adjusting the length of the reference arm using a scanning mirror (SM), only the sample wave having the same time of flight as that of the reference wave was selectively acquired. The optical band-pass filter (Thorlabs, two-photon: 510/42, SHG: 445/45) and the PMT (Hamamatsu, R7600U-300) were positioned behind the short-pass dichroic mirror for two-photon fluorescence and second-harmonic generation microscopy.

# Supplementary Note 2: Time-resolved complex electric-field imaging for focused illumination


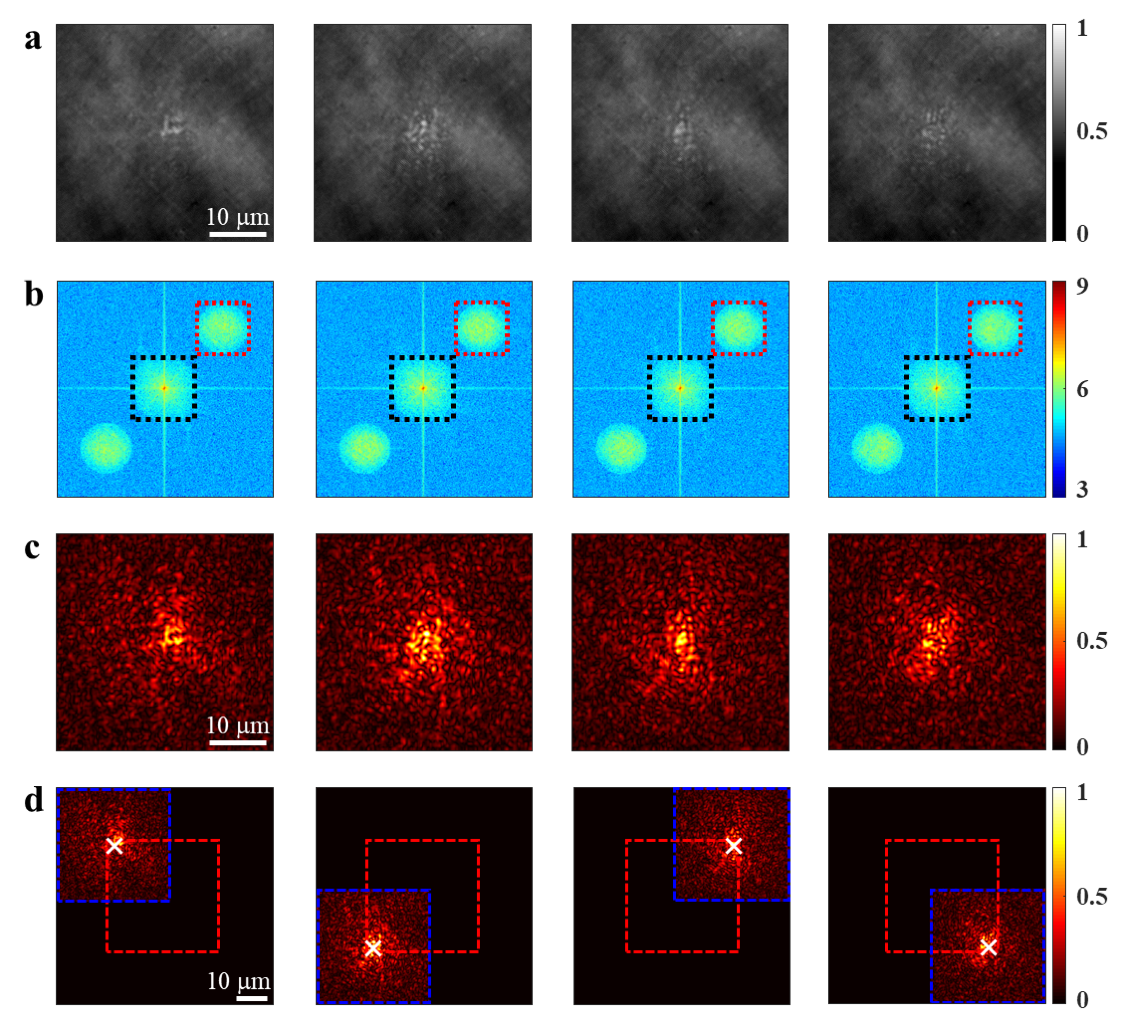


**Supplementary Figure 2. Acquisition of time-gated complex-field images. a** Raw interferometric images recorded at the camera for four different positions of illumination in the case of aberration samples. **b** Two-dimensional Fourier transforms of the raw images in **a**. Their amplitudes are shown in a logarithmic scale. The black and red dotted boxes indicate DC and AC components, respectively. **c** Inverse Fourier transforms of the AC components in **b**, which result in the complex electric field maps. Only amplitude maps are shown here. **d** Shifting the center of each complex-field map to the corresponding illumination position at the sample plane yields a rearranged field at the laboratory frame. White $\boldsymbol{\times}$ mark indicates each illumination position.

For each illumination point **r**_i_, the backscattered sample wave $E_{\mathrm{sam}}(\mathbf{r}_{\mathrm{cam}};\mathbf{r}_{i})$ is interfered with the reference planar wave $E_{\mathrm{ref}}\left( \mathbf{r}_{\mathrm{cam}} \right)=E_{r}e^{i\mathbf{k}_{r}\cdot\mathbf{r}_{\mathrm{cam}}}$ to form an interferogram at the camera. The recorded interference pattern shown in Supplementary Figure 2a is written as

$I_{\mathrm{cam}}\left( \mathbf{r}_{\mathrm{cam}};\mathbf{r}_{i} \right)=\left| E_{\mathrm{ref}}\left( \mathbf{r}_{\mathrm{cam}} \right)+E_{\mathrm{sam}}(\mathbf{r}_{\mathrm{cam}};\mathbf{r}_{i}) \right|^{2}$. (S1)

The sample wave $E_{\mathrm{sam}}(\mathbf{r}_{\mathrm{cam}};\mathbf{r}_{i})$ can be expressed as

$E_{\mathrm{sam}}\left( \mathbf{r}_{\mathrm{cam}};\mathbf{r}_{i} \right)=E_{S}\left( \mathbf{r}_{\mathrm{cam}};\mathbf{r}_{i}, \tau=\tau_{0} \right)+E_{M}\left( \mathbf{r}_{\mathrm{cam}};\mathbf{r}_{i}, \tau=\tau_{0} \right)+E_{\mathrm{DC}}\left( \mathbf{r}_{\mathrm{cam}};\mathbf{r}_{i}, \tau\neq\tau_{0} \right).$ (S2)

Here, $E_{S}$ and $E_{M}$ represent a single-scattered wave and a time-gated multiple-scattered wave, respectively, whose arrival time $\tau$ is the same as that of the reference wave $\tau_{0}$. In our main text, we defined $E_{\mathrm{cam}}\left( \mathbf{r}_{\mathrm{cam}};\mathbf{r}_{i} \right)\equiv E_{S}\left( \mathbf{r}_{\mathrm{cam}};\mathbf{r}_{i}, \tau=\tau_{0} \right)+E_{M}\left( \mathbf{r}_{\mathrm{cam}};\mathbf{r}_{i}, \tau=\tau_{0} \right)$. $E_{\mathrm{DC}}$ describes multiple-scattered waves with flight time $\tau\neq\tau_{0}$, which do not interfere with the reference wave. By taking the Fourier transform of each interference image in the spatial frequency domain, the spatial frequency spectrum of the sample’s wave is separated from the DC spectral components (Supplementary Figure 2b). By cropping the AC component and taking its inverse Fourier transform, the complex electric-field map of the sample wave reflected from the target $E_{\mathrm{cam}}\left( \mathbf{r}_{\mathrm{cam}};\mathbf{r}_{i} \right)$ is obtained in the camera coordinate (Supplementary Figure 2c). Only the amplitude map of the sample wave is shown here. The recorded sample wave field $E_{\mathrm{cam}}$ is composed of $E_{S}$ and $E_{M}$, and its broadened amplitude profile larger than the diffraction-limited spot is due to the sample-induced wavefront aberrations of single-scattered waves and time-gated multiple-scattered waves. Since the reflected sample wave was de-scanned by the Galvo mirror, the center of the sample wave was stationary upon the scanning of the focus. To find the input and output aberrations independently using the CLASS algorithm, the field must be represented in the laboratory frame with the position vector, $\mathbf{r}_{\mathrm{cam}}=\mathbf{r}_{o}-\mathbf{r}_{i}$. A time-gated sample wave in laboratory coordinate $E_{\mathrm{lab}}\left( \mathbf{r}_{o};\mathbf{r}_{i} \right)$ is constructed by laterally translating the center of the electric-field $E_{\mathrm{cam}}\left( \mathbf{r}_{\mathrm{cam}};\mathbf{r}_{i} \right)$ to the corresponding illumination position; ${E_{\mathrm{lab}}\left( \mathbf{r}_{o};\mathbf{r}_{i} \right)=E}_{\mathrm{cam}}\left( \mathbf{r}_{o}-\mathbf{r}_{i};\mathbf{r}_{i} \right)$ (Supplementary Figure 2d).

# Supplementary Note 3: Imaging geometry and coordinate systems


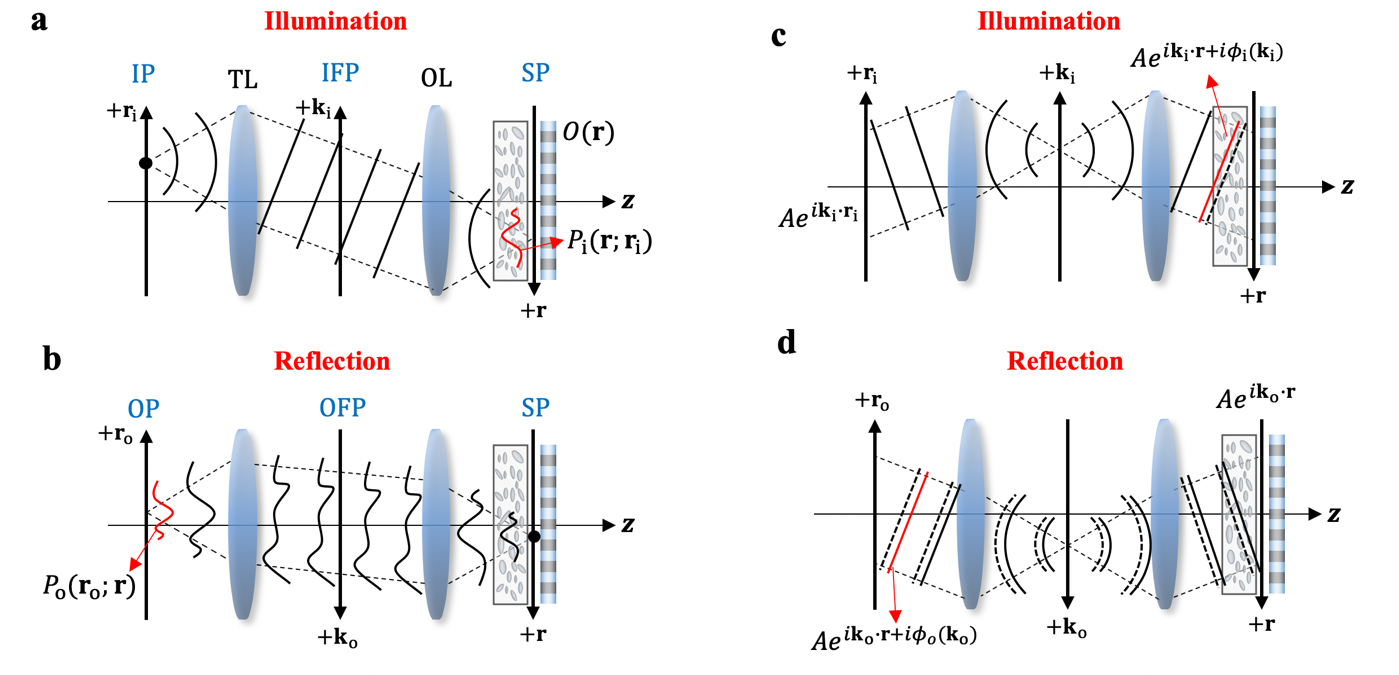


**Supplementary Figure 3. Imaging geometry and coordinate system.** **a** and **b** Space-domain description of illumination and reflection pathways, respectively. IP: input plane, IFP: input Fourier plane, SP: sample plane, OFP: output Fourier plane, OP: output plane. TL and OL represent a tube lens and an objective lens, respectively. $P_{i}\left( \mathbf{r};\mathbf{r}_{i} \right)$ is an illumination PSF in the sample plane for a point source illuminating at $\mathbf{r}_{i}$. $P_{o}\left( \mathbf{r}_{o};\mathbf{r} \right)$ is the detection PSF at the output plane for a point source leaving at $\mathbf{r}$ in the sample plane. Sample plane coordinate $\mathbf{r}$ is reversed with respect to the input plane coordinate, $\mathbf{r}_{i}$. Each lens is described as a forward Fourier transform operator, $\boldsymbol{F}.$ **c**, **d** Spatial frequency domain description of illumination and reflection pathways, respectively. A planar wave illumination with the wavevector $\mathbf{k}_{i}$ is focused at $\mathbf{k}_{i}$ in the input Fourier plane and experiences phase retardation $\phi_{i}\left( \mathbf{k}_{i} \right)$ in the sample plane. The plane wave with the wavevector $\mathbf{k}_{o}$ leaving the sample plane experiences phase retardation $\phi_{o}\left( \mathbf{k}_{o} \right)$ and is focused at $\mathbf{k}_{o}$ in the output Fourier plane. $\phi_{i}\left( \mathbf{k}_{i} \right)$ and $\phi_{o}\left( \mathbf{k}_{o} \right)$ constitute ${\tilde{\boldsymbol{P}}}_{i}$ and ${\tilde{\boldsymbol{P}}}_{o}$, respectively.

Ideal imaging system consists of 4-f relay lenses, and it is heuristic to describe the flow of coordinate transform along the propagation through lens system. A simplified schematic diagram of imaging geometry and coordinate systems of LS-RMM are shown in Supplementary Figure 3. In the illumination pathway (Supplementary Figure 3a), a point light source in the input plane is projected on the object plane via a 4f imaging system composed of two lenses. The two consecutive Fourier transformations by the two lenses lead to a negative magnification factor, i.e. an inversion of the image. In our coordinate system, the sample plane coordinate $\mathbf{r}$ is reversed with respect to the input plane coordinate $\mathbf{r}_{i}$ in order to have a positive magnification^1,2^. The first lens acts as a Fourier transform operator $\boldsymbol{F}$ within the passband set by the numerical aperture of the system and creates a 2D Fourier transform of the input field at the focal plane of the first lens with the associated spatial frequency $\mathbf{k}_{i}$. The second lens also performs a forward Fourier transform of the field in its focal plane into the object plane, followed by the coordinate inversion. Let us denote $\boldsymbol{\Pi}$ as an inversion (or parity) operator which changes the sign of the object coordinate: $\boldsymbol{\Pi}\mathbf{:r}\boldsymbol{\mapsto}\mathbf{-}\boldsymbol{r}$. Since $\exp\left( \boldsymbol{-}i\left( -\mathbf{r} \right)\cdot\mathbf{k}_{i} \right)=\exp\boldsymbol{(+}i\boldsymbol{r\cdot}\mathbf{k}_{i})$, a forward Fourier transform followed by a coordinate inversion is the same as an inverse Fourier transform with the positive sign in the exponentials^2^: $\boldsymbol{F}^{-1}\boldsymbol{=}\boldsymbol{\Pi}\boldsymbol{F}$. Here, we use the convention of the negative sign in the exponential for a forward Fourier transform. Similar to the illumination pathway, a wave emitted from a point in the object plane is projected back onto the output plane (Supplementary Figure 3b). The reflection matrix of the whole imaging system including a target sample in position basis is obtained by multiplying all the transfer matrices sequentially:

$$\boldsymbol{R}\boldsymbol{=\Pi FF}\boldsymbol{P}_{o}\boldsymbol{O}\boldsymbol{P}_{i}\boldsymbol{\Pi FF}$$

$$\boldsymbol{=}\boldsymbol{F}^{-1}\boldsymbol{F}\boldsymbol{P}_{o}\boldsymbol{O}\boldsymbol{P}_{i}\boldsymbol{F}^{-1}\boldsymbol{F}$$

$\mathbf{=}\boldsymbol{P}_{o}\boldsymbol{O}\boldsymbol{P}_{i}.$ (S3)

This relation shows how we record the space-domain reflection matrix in the input and output planes set by 4-f relay system. The reflection matrix in spatial frequency basis, $\mathbf{k}_{i}$ and $\mathbf{k}_{o}$, is given by

$$\tilde{\boldsymbol{R}}\boldsymbol{=F}\boldsymbol{P}_{o}\boldsymbol{O}\boldsymbol{P}_{i}\boldsymbol{\Pi F}$$

$$\boldsymbol{=F}\boldsymbol{P}_{o}\boldsymbol{O}\boldsymbol{P}_{i}\boldsymbol{F}^{\boldsymbol{-1}}$$

$\boldsymbol{=FR}\boldsymbol{F}^{\boldsymbol{-1}}\mathbf{.}$ (S4)

This relation, i.e. the sequence of operations $\boldsymbol{F}^{\boldsymbol{-1}}$🡪$\boldsymbol{R}\boldsymbol{F}$ is described in Supplementary Figure 3c and 3d. A planar wave with $\mathbf{k}_{i}$ is leaving as a point source at the input Fourier plane. It is inversely Fourier transformed by the objective lens and coordinate inversion to be a planar wave $e^{i\mathbf{k}_{i}\boldsymbol{\cdot}\mathbf{r}}$ at the sample plane (Supplementary Figure 3c). This incident wave is reflected by the target sample including scattering medium, which is described by the multiplication of the space-domain reflection matrix $\boldsymbol{R}$. A representative planar wave with $\mathbf{k}_{o}$ constituting the reflected wave is focused at the output Fourier plane by the Fourier transform of the OL in the reflection pathway (Supplementary Figure 3d).

# Supplementary Note 4: Detector’s dynamic range in multiple-scattering regime


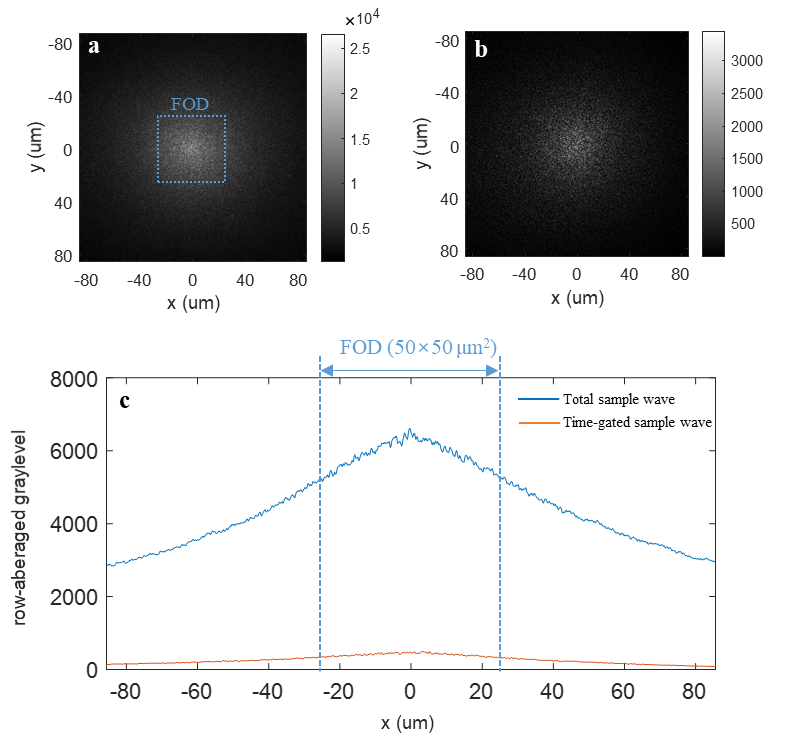


**Supplementary Figure 4. The intensity distribution of the sample wave in strong multiple scattering regime.** **a** Intensity image of the sample wave backscattered from mouse brain through a 150-μm-thick intact skull. The imaging depth is about 50 μm below the cortical surface. The blue-dotted box indicates a typical FOD of 50×50 μm^2^, in which the aberrated single-scattered waves are expected to be localized. **b** Intensity image of the time-gated sample wave, obtained from off-axis digital holography. The color bars in **a** and **b** indicate the gray levels of the camera pixels. The intensity of the reference field used here was about 25,000 in gray levels. **c** Row-averaged intensity profiles of the sample wave (blue line) and the time-gated sample wave (red line). The backscattered sample wave consists of a significant contribution of the non-interfering multiple-scattered wave $E_{\mathrm{DC}}$, which acts like a DC background noise and predominantly saturate the camera.

In terms of detector’s dynamic range, the image-plane detection along with focus illumination becomes beneficial over the pupil-plane detection as multiple scattering noise is increased. In the case of strong multiple scattering and aberrations, the non-interfering multiple-scattered wave $E_{\mathrm{DC}}$ becomes much stronger than the single-scattered wave $E_{S}$ or the time-gated multiple-scattered wave $E_{M}$, especially when there is no bright, high-contrast object in the focal plane. Then, the camera pixels are saturated predominantly by strong multiple-scattered wave $E_{\mathrm{DC}}$ regardless of the location of the detection plane, which impedes high-dynamic-range imaging of the single-scattered wave $E_{S}$. In the pupil-plane detection, all the multiple-scattered waves collected by the objective lens uniformly fill the pupil aperture. In the image-plane detection, the collected multiple-scattered waves spread over the full field of view of the objective lens (FOV_obj_), which is much larger than our FOD at the camera. FOV_obj_ was about 250 μm in diameter, and a typical size of FOD was 50×50 μm^2^ for the through-skull imaging. Therefore, only a factor of 25 of total collected multiple scattering is detected in the image-plane detection. When we convert the image-plane basis to the pupil-plane basis for the CLASS algorithm, the single- to multiple-scattering intensity ratio is reduced by the same factor in comparison with the pupil-plane detection.

A single-shot intensity image of the sample wave backscattered from mouse brain through 150-μm-thick intact skull is shown in Supplementary Figure 4a. The illumination was focused at the depth of 50 μm below the cortical surface, and the backscattered wave was recorded by a 16-bit digital camera in a conjugate image plane. The time-gated intensity image of the sample wave (Supplementary Figure 4b) is obtained by means of digital off-axis holography with a reference plane wave. As compared in Supplementary Figure 4c, the intensity of the non-interfering multiple-scattered wave $\left| E_{\mathrm{DC}} \right|^{2}$ is one order of magnitude higher than that of the time-gated sample waves, $\left| E_{S}+E_{M} \right|^{2}$. The FOD in LS-RMM can be understood as a physical pinhole that efficiently discriminates the single-scattering (or snake-like) signal from the multiple-scattering noise in the recording stage.

# Supplementary Note 5: The size of FOD and its effect on the performance of aberration correction


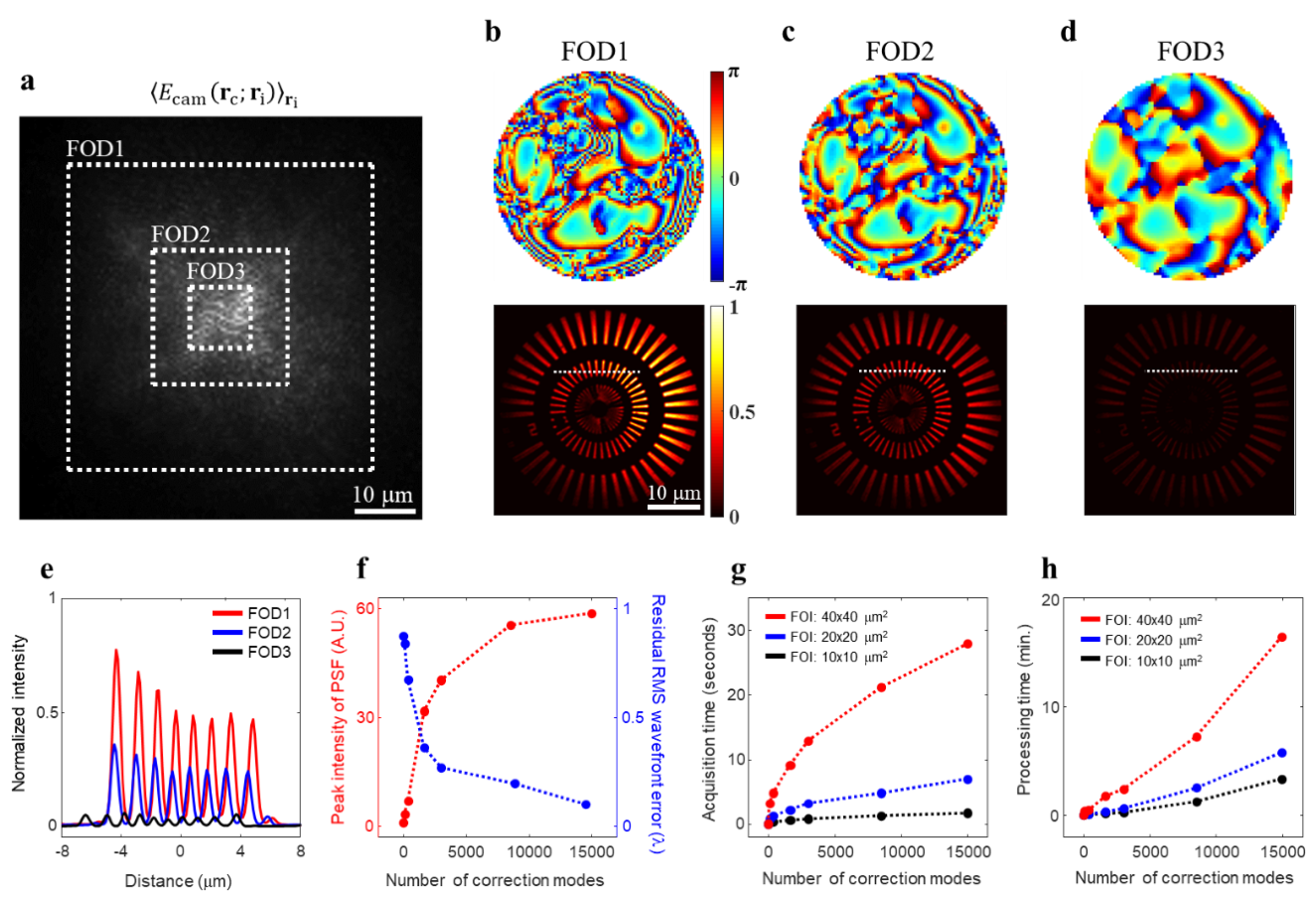


**Supplementary Figure 5. Aberration correction depending on the size of FOD.** **a** Amplitude map of $E_{\mathrm{cam}}\left( \mathbf{r}_{\mathrm{cam}};\mathbf{r}_{i} \right)$for the sample with aberrations averaged over $\mathbf{r}_{i}$ in FOI of 40$\times$40 μm^2^. White dotted boxes indicate three representative FODs. From the largest one, FOD1: 50$\times$50 μm^2^, FOD2: 22$\times$22 μm^2^, and FOD3: 10$\times$10 μm^2^. The corresponding numbers of correction modes in the pupil are 9700, 1900, and 390, respectively. **b-d** Aberration map (upper) and intensity image (lower) obtained after applying the CLASS algorithm for each FOD. Color bars for the upper and lower images are phase in radians and intensity normalized by the maximum intensity in **b**, respectively. **e** Line plots along the white dotted lines in **b-d**. **f** Peak intensity of the aberration-corrected PSF (red line) depending on the number of correction modes set by FOD. The residual RMS wavefront error (blue line) relative to the aberration map obtained at FOD: 66$\times$66 μm^2^ corresponding to the number of correction modes of 17,000. **g**, **h** Data acquisition time and processing time depending on the number of correction modes set by FOD, respectively.

In our system, the FOI is defined by the scanning area of the focused illumination, while the FOD is defined by the detection area at the camera. Therefore, FOD can be chosen independently of FOI depending on the spread of PSF. An averaged intensity image of the reflected electric-field map $E_{\mathrm{cam}}\left( \mathbf{r}_{\mathrm{cam}}\mathbf{;}\mathbf{r}_{i} \right)$ for various illumination positions $\mathbf{r}_{i}$ is shown in Supplementary Figure 5a. White dotted boxes show three representative FODs, i.e., FOD1: 50$\times$50 μm^2^, FOD2: 22$\times$22 μm^2^, and FOD3: 10$\times$10 μm^2^. The number of correction modes for FOD1, FOD2, and FOD3 are given as 9700, 1900 and 390, respectively. Note that FOD1 covers almost all the spread of PSF. On the contrary, smaller FOD misses some of the broadened PSF due to aberrations, meaning that aberration correction will be incomplete and high frequency component of the aberration map cannot be measured. The aberration maps gradually lost fine details as the FOD is reduced, and the contrast and spatial resolving power of the reconstructed images were reduced as a consequence (Supplementary Figures 5b-d). This is confirmed by the line plots along the white dotted lines in Supplementary Figures 5b-d shown in Supplementary Figure 5e. For further characterization, we plotted the peak intensity of the aberration-corrected PSF (red dots) and residual root-mean-square (RMS) wavefront error (blue dots) as functions of the number of correction modes set by the size of FOD (Supplementary Figures 5f). The residual RMS wavefront errors were measured relative to the aberration map acquired with FOD of 66$\times$66 μm^2^, which correspond to the number of correction modes of 17000. As the larger number of correction modes is used, the residual RMS wavefront error becomes smaller and consequently the Strehl ratio is enhanced. However, the use of larger FOD is not always beneficial because data acquisition and image processing times become longer with the increase of the FOD (Supplementary Figures 5g and 5h). These are more pronounced as the FOI is increased. Therefore, the careful choice of the FOD is required to ensure both the optimal aberration corrections and data acquisition/image processing times.

# Supplementary Note 6: A new algorithm for correcting spatially varying high order aberrations

Scattering medium such as a mouse skull consists of fine inhomogeneous structures over several layers. Not only does this cause complex optical aberrations, but it makes the isoplanatic patch size so small that a single aberration correction map cannot properly handle locally varying aberrations. To obtain object image over the wide field of view, it is necessary to analyze several subregions separately. In conventional computational AO and the previous CLASS algorithm, dividing the entire FOI into small subregions accompanies the simultaneous reduction of FOI and FOD in each subregion. This results in the reduced the number of correction modes, making it difficult to correct complex aberrations in small isoplanatic patches. The entire FOI was divided into several subregions (white dashed boxes) in the conventional imaging to find local aberrations (Supplementary Figure 6a). The FOI (red box: 10 × 10 µm^2^) and FOD (blue box: 10 × 10 µm^2^) at each subregion were reduced accordingly. Figure S6b shows a set of 16 reflection matrices constructed for individual subregions. Each matrix is square with the number of columns (rows) set by the number of modes in the reduced FOI (FOD), which was 390. The CLASS algorithm was applied to each matrix in Supplementary Figure 6b. CLASS images and aberration maps were obtained for individual subregions and merged together as shown in Supplementary Figures 6c and 6d, respectively. Due to the reduced number of correction modes in each subregion, the myelinated axons were invisible.


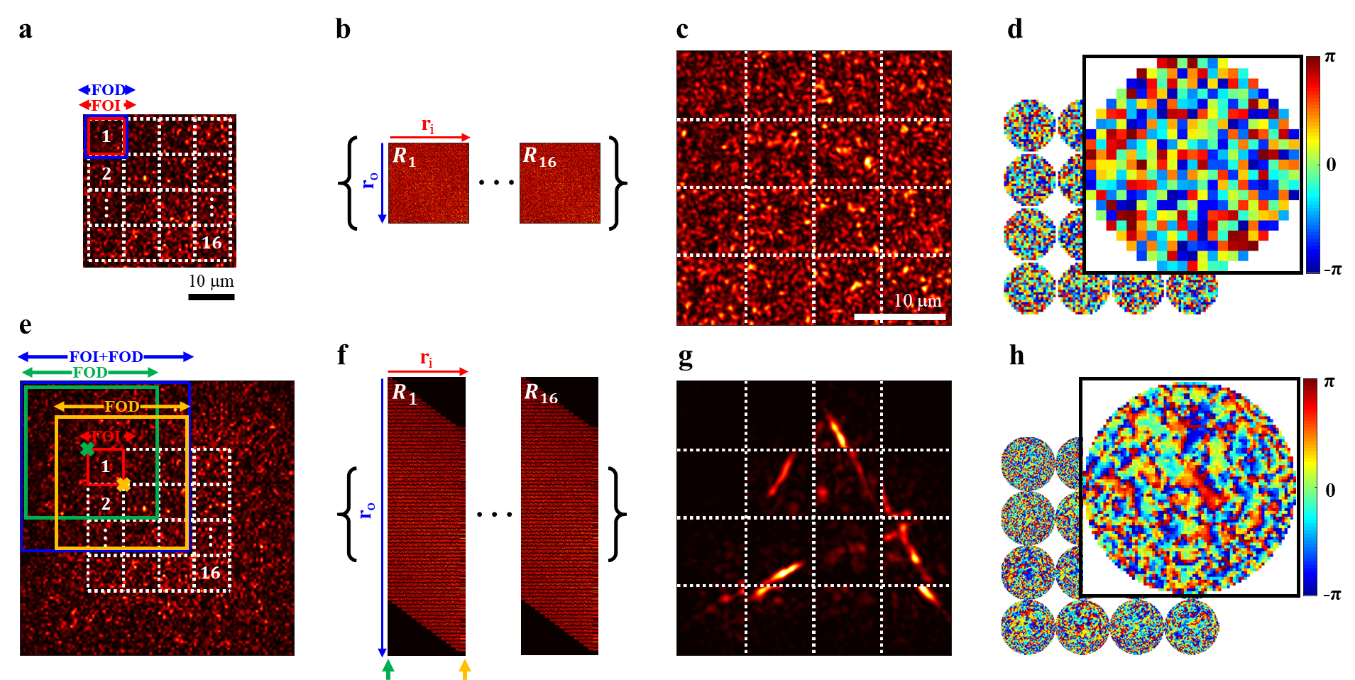


**Supplementary Figure 6. Aberration correction algorithm for correcting a large number of modes for small isoplanatic patches. a** Conventional imaging where FOI and FOD are the same. The white dashed lines indicate that the entire FOI area is divided into 4$\boldsymbol{\times}$4 small areas. The total FOI is 30 × 30 µm^2^, and the size of each subregion is 10 × 10 µm^2^ including the margin. The number in each small white dashed square labels each subregion. To analyze local aberration for subregion #1, for example, the FOI and FOD are chosen as red and blue squares, respectively. **b** Reflection matrix acquired for each subregion. Since the sizes of the FOI and the FOD are the same, 16 square matrices can be obtained for individual subregions. The number of columns (rows) is given by FOI (FOD). **c**, **d** CLASS image and corresponding aberration map, respectively, for each subregion after applying CLASS algorithm to each matrix in **b**. The number of correction modes per patch in **d** is 390. **e** LS-RMM imaging for the same FOI of 30 × 30 µm^2^ (dashed white square) as the conventional imaging. The image shows the coherent addition of $E_{\mathrm{lab}}\left( \mathbf{r}_{o};\mathbf{r}_{i} \right)$ for $\mathbf{r}_{i}$ covering the FOI. For each subregion, the FOI area is set to be the same as **a** (red box: 10 × 10 µm^2^), but the detection is made for a larger area of FOD (30 × 30 µm^2^). The green and yellow boxes indicate FODs for the two representative illumination points indicated by the green and yellow × marks, respectively. The blue square indicates the total detection area for the given FOI in the patch #1. **f** Reflection matrix was constructed for each subregion. Each matrix is rectangular because the number of columns is determined by the FOI, while the number of rows is determined by the convolution of the FOI and FOD (blue box in **e**: 40 × 40 µm^2^). The green and yellow arrows point to the columns obtained from the green and yellow boxes in **e**, respectively. **g,** **h** CLASS image and aberration maps, respectively, using each matrix in **f**. The number of correction modes per patch in **h** is given by FOD, which was 3500.

In the LS-RMM, even though the FOI is reduced to the subregion (red box in Supplementary Figrue 6e: 10 × 10 µm^2^), we can set FOD independently of FOI in such a way that the information outside the subregion can be made use of. In our experiment, we set the FOD (green and yellow box: 30 × 30 µm^2^) wider than FOI to measure and correct high order aberrations. In order to utilize the field outside the patch without losing the measured information, the number of rows in the matrix corresponding to each subregion must be determined by the convolution of the FOI and FOD sizes (blue box: 40 × 40 µm^2^). Therefore, the reflection matrix for each subregion becomes rectangular (Supplementary Figure 6f). In our advanced CLASS algorithm, we first identified aberration maps $\phi_{o}\left( \mathbf{k}_{o} \right)$ for 3,500 modes in the rows of the matrix, which is set by FOD, and applied this to $\phi_{i}\left( \mathbf{k}_{i} \right)$ in the 390 columns after matching the number of columns with the number of rows by the interpolation. By repeating this process, we can correct aberrations for 3500 angular modes set by FOD. In this way, the number of correction modes is maintained when the FOI is reduced to cope with the small isoplanatic patch. Due to this capacity, we could identify locally varying high order aberrations (Supplementary Figure 6h) and reconstruct clear myelinated axons (Supplementary Figure 6g) through an intact skull.

# Supplementary Note 7: PSF comparison before and after aberration correction for through-skull imaging


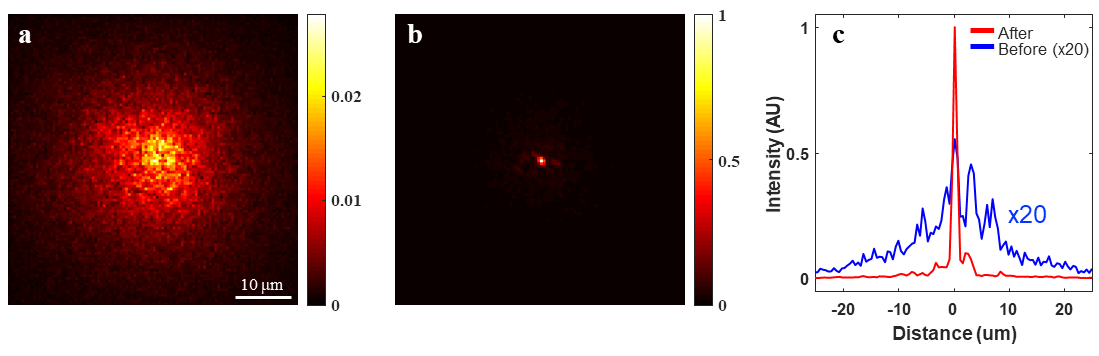


**Supplementary Figure 7. PSFs before and after aberration correction for the through-skull imaging.** **a** Intensity PSF before aberration correction. **b** Intensity PSF after aberration correction by CLASS algorithm. The color bars are normalized by the maximum value in **b**. **c** Line profiles of PSFs before (blue) and after (red) aberration correction. The PSF intensity before aberration correction is enlarged by 20 times for visibility.

Supplementary Figure 8 shows the intensity PSFs before and after aberration correction obtained from the through-skull imaging of 8-week-old mouse brain in Fig. 3e-h. The width of PSF broadened due to aberration and multiple scattering was narrowed down by 15 times to form a near-diffraction-limited PSF after the aberration correction. The peak intensity at the center of the PSF was increased by about 30 times.

Let us explain the effect of aberration correction to the PSF in detail. The PSF before aberration correction consists of the aberrated single-scattered wave and time-gated multiple-scattered wave, i.e. $E_{\mathrm{lab}}\left( \mathbf{r}_{o};\mathbf{r}_{i} \right)=E_{S}\left( \mathbf{r}_{o};\mathbf{r}_{i}, \tau=\tau_{0} \right)+E_{M}\left( \mathbf{r}_{o};\mathbf{r}_{i}, \tau=\tau_{0} \right)$. After the aberration correction, the aberrated single-scattered wave is focused back to $\mathbf{r}_{o}=\mathbf{r}_{i}$, the original illumination spot. If we denote the Strehl ratio enhancement of the single-scattered wave as $\alpha_{s}$, then aberration corrected field can be written as $E_{\mathrm{lab}}^{c}\left( \mathbf{r}_{o}=\mathbf{r}_{i};\mathbf{r}_{i} \right)=\sqrt{\alpha_{s}}E_{S}\left( \mathbf{r}_{i};\mathbf{r}_{i}, \tau=\tau_{0} \right)+E_{M}\left( \mathbf{r}_{i};\mathbf{r}_{i}, \tau=\tau_{0} \right)$. In our experiment, we can estimate $\alpha_{s}$ from the aberration maps identified by our CLASS algorithm. In the case of skull imaging (Fig. 3h), $\alpha_{s}$ is estimated to be about 400. Therefore, the increase of peak intensity after the aberration correction is written as

$\alpha_{S+M}=\frac{\alpha_{s}\left| E_{S} \right|^{2}+\left| E_{M} \right|^{2}}{\left| E_{S} \right|^{2}+\left| E_{M} \right|^{2}}.$ (S5)

In our experiment in Supplementary Figure 8c, $\alpha_{S+M}$ was measured to be about 30. Therefore, $\frac{\left| E_{S} \right|^{2}}{\left| E_{M} \right|^{2}}\approx\frac{\alpha_{S+M}}{\alpha_{s}-\alpha_{S+M}}$ was about 0.08, which means that single-scattered wave was initially 10 times weaker than the time-gated multiple-scattered waves.

The enhancement of the Strehl ratio is often used as a measure of the performance of an AO system because it indicates the increase of reconstructed image intensity after the aberration correction. In the weak multiple scattering, the Strehl ratio enhancement is determined by $\alpha_{s}$. On the contrary, it is measured to be $\alpha_{S+M}\left( \ll\alpha_{s} \right)$ as multiple scattering is significantly larger than the single scattering. Therefore, even if the aberrated single-scattered wave is well focused back to the confocal point, the effective Strehl ratio enhancement, $\alpha_{S+M}$, appears to be much lower due to the presence of multiple scattering noise.

# Supplementary Note 8: Systematic search for the isoplanatic patch size


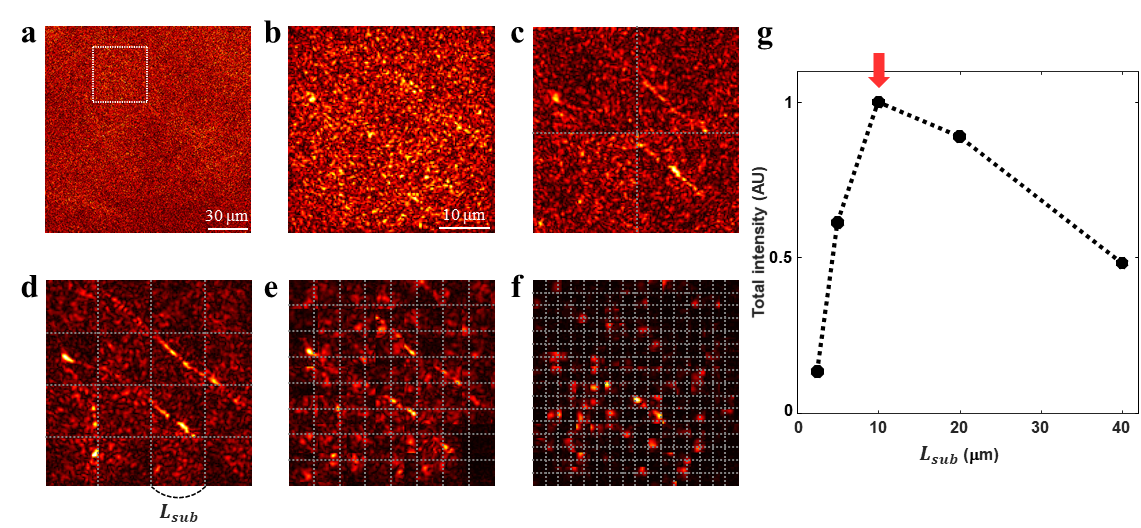


**Supplementary Figure 8. Systematic search for the isoplanatic patch size.** **a** Conventional OCM imaging through the intact skull of an 8-week-old mouse shown in Fig. 3f. **b-f** CLASS images obtained by varying the size of the subregion ($L_{\mathrm{sub}}$) for a 40×40 µm^2^ indicated by the white box of **a**. $L_{\mathrm{sub}}$’s corresponding to **b-f** are 40 μm, 20 μm, 10 μm and 5 μm, 2.5 μm, respectively. **g** Graph showing the relationship between $L_{\mathrm{sub}}$ and total intensity of **b-f**. The point with the largest total intensity indicated by the red arrow corresponds to **d**,$L_{\mathrm{sub}}$ = 10 μm.

We can systematically find the optimal subregion size by monitoring the total intensity of the CLASS image. The CLASS algorithm is intended to constructively accumulate non-confocal signal back to confocal points. If it works well, then the total intensity in the reconstructed image is to be increased. To make this point clear, we chose 30×30 µm^2^ area in the intact skull imaging in Fig. 3f, which is shown as dotted box in Supplementary Figure 7a, and applied CLASS algorithm to the area while reducing the size of subregion (Supplementary Figures 6b-f) and plotted the total intensity of the CLASS image as a function of the size of subregion in Supplementary Figure 6d. We found that the total intensity is maximum when the subregion size is 10×10 µm^2^, where the filamented structures of myelinated axons are clearest.

# Supplementary Note 9: SHG imaging through an intact mouse skull by the physical aberration correction

We demonstrated near-diffraction-limited SHG imaging through an intact skull. We placed collagen gel matrix under a 100-μm-thick mouse skull excised from a 3-week-old mouse (Supplementary Figure 9a). Collagen gel was made from rat tail collagen (Collagen type I, Corning, New York, USA). Collagen type I gel solution (2 mg/mL) was neutralized with 0.1 N NaOH and chilled with ice. The mixed solution was incubated at 37°C for 60 min to form the collagen gel. Skulls were excised from 3-week-old C57BL/6 mice and promptly immerged in PBS. Skulls fixed with 4% paraformaldehyde were washed with PBS and mounted on the collagen gel for imaging. The objective focus was set 240 μm below the bottom surface of the skull. Due to complex aberrations by the skull, conventional OCM failed to visualize any collagen fibers (Supplementary Figure 9b). The entire field of view was divided into 6×6 subregions (each subregion size is about 12×12 μm^2^ including the overlap with adjacent areas), and the CLASS algorithm was applied independently to each subregion (Supplementary Figure 9c). The fine collagen fibrils were clearly resolved. The number of correction modes in each aberration map was about 10,000 (Supplementary Figure 9d).


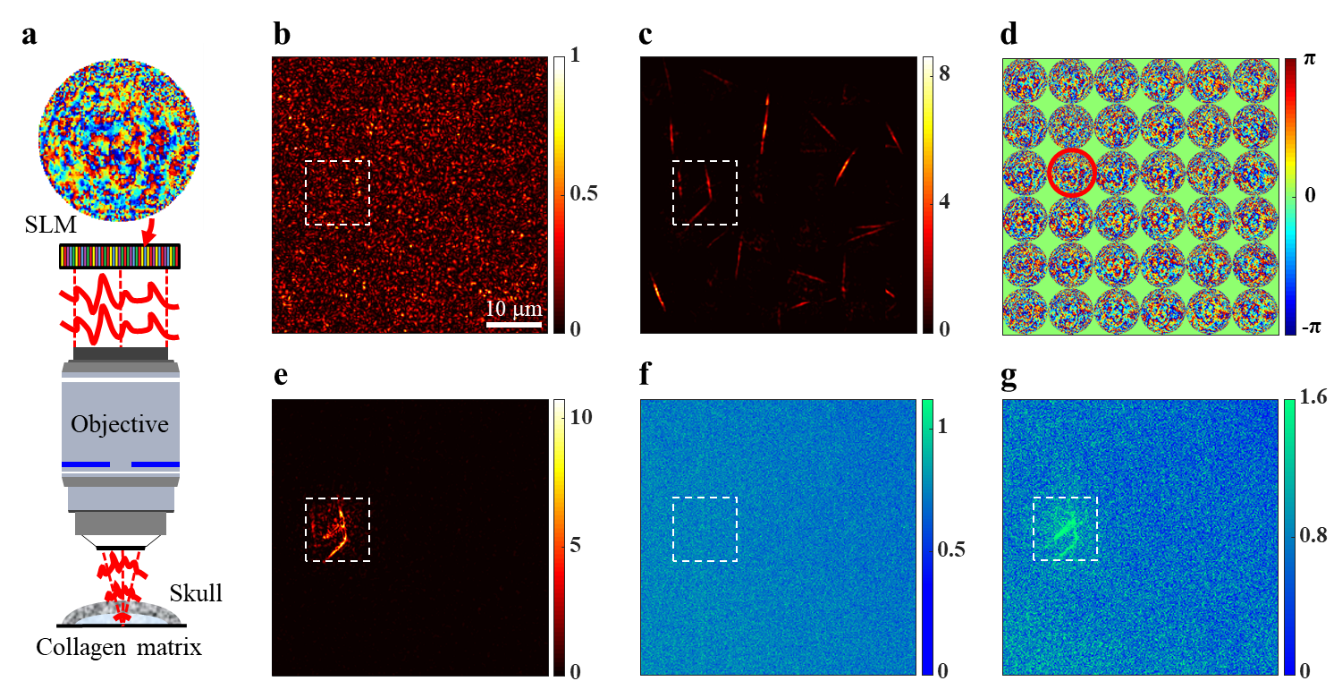


**Supplementary Figure 9. SHG imaging of collagen fibers through an intact mouse skull.** **a** Experimental schematic of hardware correction of skull-induced aberrations and SHG microscopy. The mouse skull excised from a 3-week-old mouse was placed on top of collagen gel matrix. The thickness of the skull was about 100 μm. **b** Conventional OCM image without aberration correction. Scale bar, 10 μm. **c** LS-RMM image stitched after applying aberration correction to each of 6$\boldsymbol{\times}$6 subregions. **d** Pupil phase maps for the individual subregions. **e** OCM image obtained after physically correcting the pupil aberration indicated by the red circle in **d** by using the SLM. **f** and **g** SHG images before and after physically correcting the pupil aberration by using the SLM, respectively. The white dashed box in each figure indicates the subregion where the physical aberration correction was applied. Color bars in **b, c** and **e** indicate intensity normalized by the maximum intensity in **b**. Color bars in **d**, phase in radians.

Similar to the demonstration in Figs. 2j-m, we could physically correct the skull-induced aberrations by displaying the phase conjugation of the aberration map on the SLM. Since the aberrations varied depending on the position, we made a physical correction for each subregion at a time. For example, we chose the aberration map indicated by the red circle in Supplementary Figure 9d, which corresponds to the area in white dashed box in each image in Supplementary Figure 9, and its phase conjugation was written on the SLM. A conventional OCM image was taken again after hardware correction of the local aberration (Supplementary Figure 9e). Only the collagen fiber structures associated with the area indicated by the red circle were resolved. No structures were visible in the other areas, supporting that the isoplanatic size was as small as ~12$\times$12 μm^2^. Simultaneously, we took SHG images before and after applying hardware correction by SLM (Supplementary Figures 9g and 9f, respectively). While no fiber structures were visible at all in the uncorrected SHG image, the same fiber structures as those resolved by aberration-corrected OCM image were seen in the aberration-corrected SHG image. The measured width of collagen fiber was as small as 500 nm, close to the diffraction limit.

# Supplementary References

1. Jürgen Jahns and Stefan Helfert. Chapter 4.8.1 4f setup. in *Introduction to Micro- and Nanooptics* 448 (Wiley, 2012).

2. Harrison H. Barrett and Kyle J. Myers. Chapter 9.7.2 4f imaging system. in *Foundations of Image Science* (Wiley, 2004).
